# Storytelling as a transformative tool: how narrative-based teaching reshapes clinical education outcomes in vocational nursing internships

**DOI:** 10.3389/fmed.2025.1587526

**Published:** 2025-06-18

**Authors:** Le Xiao, Deqing Peng, Jindan Zhong, Tanglin Fang, Lei Dai, Xuyun Peng, Chengying Yu, Feiyan Huang

**Affiliations:** ^1^School of Medical and Nursing, Xuancheng Vocational and Technical College, Xuancheng, Anhui, China; ^2^Xuancheng People’s Hospital, Xuancheng, Anhui, China

**Keywords:** narrative pedagogy, clinical competency, vocational nursing education, professional identity, reflective practice

## Abstract

**Background:**

Traditional clinical apprenticeships in Chinese vocational nursing education often inadequately address professional identity development. This study evaluates the effectiveness of narrative pedagogy in enhancing professional identity and clinical competencies among vocational nursing interns.

**Objective:**

To evaluate the impact of narrative teaching interventions on two primary outcomes: professional identity development and clinical competency acquisition among vocational nursing interns.

**Methods:**

Using stratified cluster randomization, 82 clinical interns from a tertiary hospital were allocated to either: Observation group (*n* = 42) received biweekly narrative teaching sessions incorporating storytelling, role-playing, reflective writing, and clinical application cycles. Control group (*n* = 40) standard one-on-one apprenticeship training. Post-intervention evaluations utilized the Nurse Professional Identity Scale, the Nursing Intern Core Competency Scale, and a custom teaching evaluation form.

**Results:**

The observation group significantly outperformed the control group in professional identity and core competency dimensions (*P* < 0.001), with notable improvements in personal traits and clinical skills. They also excelled in learning interest, teamwork, and clinical thinking (*P* < 0.05).

**Conclusion:**

Narrative-based teaching enhances vocational nursing students’ professional identity, competencies, and internship quality, offering a valuable model for emotional and professional development.

## 1 Introduction

Clinical practicum serves as the critical transition from theoretical knowledge to clinical competence in vocational nursing education, yet significant gaps remain in equipping students to address contemporary healthcare complexities (1). In China, where vocational programs supply over 70% of frontline healthcare workers National Health Commission, the traditional “one-on-one” mentorship model faces mounting criticism. Studies reveal that 65% of interns report inadequate ethical decision-making guidance, while 58% lack professional identity development opportunities (2, 3). These challenges are exacerbated by overcrowded clinical placements (mentor-to-student ratio 1:18) and curricula prioritizing procedural repetition over reflective practice (4, 5). Consequently, graduates excel technically but struggle with holistic care—a disconnect magnified by China’s aging population (6).

Narrative pedagogy, rooted in Mezirow’s transformative learning theory, bridges cognitive and affective domains through storytelling. Two frameworks dominate: the instrumental model (curating clinical cases for knowledge acquisition) (7–9) and the experiential-inquiry model (transforming perspectives through lived experiences) (10, 11). Globally, randomized trials show 22% higher clinical decision-making confidence (12) and 18% improved diabetes management skills with narrative methods. However, 89% of evidence originates from Western baccalaureate programs (13), neglecting vocational systems in resource-limited settings like China.

The adoption of narrative pedagogy in China remains fragmented. Although urban hospitals have successfully implemented case-based narratives (14), rural colleges—which train 60% of nurses—face persistent challenges in scalability and cultural compatibility (15). Key barriers include: Cultural incongruence: western individual reflection conflicts with Confucian group harmony; Scalability gaps: virtual tools lack professional identity integration; Ethical training deficits: only 32% of curricula address narrative ethics in complex care. To address these challenges, we developed the Experience-Inquiry Narrative Model (EINM) grounded in Schön’s reflective practice theory: Co-constructed storytelling: mentor–student analysis of ethical dilemmas (e.g., end-of-life conflicts); Role-embodied simulation: VR-based scenario reenactment; Collective debriefing: guided discussions aligning experiences with Confucian social responsibility. This mixed-methods study examines: narrative pedagogy’s role in transitioning from technical skill to professional identity; cultural adaptations for scalable implementation in low-resource settings.

Preliminary RCT data (*n* = 120) show EINM participants achieved: 17.3% higher professional identity scores (NPIS-21, α = 0.89; *P* < 0.01); 24% improvement in clinical judgment accuracy during standardized case analyses (*P* < 0.05); 31% improved patient communication evaluations. By harmonizing global evidence with China’s cultural realities, this research pioneers a scalable framework to transform vocational nurses from task technicians to reflective practitioners.

## 2 Subjects and methods

### 2.1 Research subjects

A convenience sampling method was employed to recruit 82 full-time nursing interns from full-time medical higher vocational college or Vocational and Technical College during their clinical rotations at a tertiary-grade A hospital from August 2023 to April 2024. Sample size calculation was performed using G*Power 3.1 with the following parameters: Effect size: Cohen’s *d* = 0.7 (based on prior studies of narrative pedagogy (14)); α error probability: 0.05; Power (1−β): 0.80. The initial calculation yielded a minimum requirement of 78 participants (39 per group). To account for clustering effects from departmental rotation groupings, we adjusted the sample size using the formula: *N*_adjusted_ = _N÷(1–ICC)_. Where the intraclass correlation coefficient (ICC) was set to 0.15, consistent with values reported in nursing education cluster trials (16). This adjustment resulted in a final sample of 82 participants.

Inclusion criteria:

1. Enrolled in a full-time nursing program;
2. Commitment to attend ≥90% of intervention sessions;
3. Willingness to complete narrative assignments and engage in reflective discussions.

Exclusion criteria:

1. Cumulative absence ≥1 month during the internship;
2. Missed ≥2 scheduled interventions due to personal reasons;
3. Active withdrawal or leave of absence from the program.

Ethical considerations:

To minimize confounding, participants were stratified by school affiliation and residential district and grouped into 16 clusters (14 clusters of 5 interns and 2 clusters of 6 interns). Each cluster underwent sequential rotations through eight departments (e.g., internal medicine and surgery) with no inter-cluster contact. Allocation concealment was ensured via computer-generated block randomization (block size = 4), assigning eight clusters to the intervention group (*n* = 42) and eight clusters to the control group (*n* = 40).

In the observation group, there were 5 male and 37 female participants, with an average age of 20.40 ± 0.587 years. The average score for basic nursing courses was 76.86 ± 10.363. Of the participants, 30 were from Anhui Province, while 12 were from other provinces. In the control group, there were 6 male and 34 female participants, with an average age of 20.63 ± 0.586 years. The average score for basic nursing courses was 76.18 ± 7.445. Of the participants, 34 were from Anhui Province, while 6 were from other provinces. There were no statistically significant differences between the two groups in terms of gender, age, nursing course scores, or place of origin (*P* > 0.05).

### 2.2 Methods

#### 2.2.1 The control group used traditional teaching methods

The control group underwent a standardized clinical apprenticeship aligned with national standards for nursing education. Participants were paired with registered nurse preceptors meeting stringent criteria: minimum 5 years of clinical experience; per the Provincial Tertiary Hospital Accreditation Standards, clinical nursing instructors must pass the hospital’s 40-h standardized training program with excellence grades (≥90/100) in both theoretical and skills assessments; preceptors’ teaching quality was monitored using the hospital’s standardized 5-point mentoring evaluation rubric (technical instruction 30%, feedback timeliness 30%, and safety supervision 40%). The 8-month program included:

*Weekly skill sessions:* Structured clinical training comprised weekly 1.5-h skill demonstrations supervised by staff nurses, covering six fundamental procedures (e.g., vital sign measurement and aseptic technique) as per the Nursing Internship Training Guidelines.

*Monthly case discussions:* 1.5-h rounds analyzing real patient cases from hospital records;

*Competency evaluation:* Final assessments using the hospital’s standardized Objective Structured Clinical Examination (OSCE) stations, covering: technical skills (e.g., sterile technique and medication safety); clinical judgment (case scenario analysis); communication skills (standardized patient interactions). Assessments were conducted by a panel of three senior nurses (average working years 15.2 ± 3.8 years) using validated rubrics with established reliability (Cronbach’s α = 0.89 in pilot testing).

#### 2.2.2 The observation group adopted a narrative-based teaching model

The intervention group received a narrative-enhanced clinical education program over 8 months, systematically integrating storytelling into the apprenticeship model. The intervention was designed following evidence-based frameworks in nursing education and consisted of three key components:

##### 2.2.2.1 Interprofessional educator team development

A multidisciplinary teaching team was established through a three-stage selection process:

###### 2.2.2.1.1 Clinical experts

Eight Senior Nurse Practitioner (Intermediate Grade) (mean clinical experience = 12.5 ± 3.2 years) from eight clinical specialties (internal medicine, surgery, ICU, etc.), selected based on:

Minimum 5 years of bedside experience; top quartile performance in hospital teaching evaluations (Hospital Education Quality Monitoring System); completion of a 40-h hospital-based narrative pedagogy training program focused on illness experience sharing and reflective practice, with competency validation through internal assessment protocols.

###### 2.2.2.1.2 Academic faculty

Four nursing professors with: peer-reviewed publications on narrative education (≥3 first-author papers); provincial-level teaching awards in nursing education; demonstrated expertise in competency-based assessment design.

All team members underwent standardized training using Ironside’s Narrative Pedagogy Framework (10), achieving 85% inter-rater agreement on case selection criteria through structured group discussions.

##### 2.2.2.2 Contextualized narrative content development

Sixteen case modules were developed through systematic analysis of 237 clinical incident reports (2020–2022) from participating hospitals. Key thematic areas included:

Clinical decision-making (five cases focusing on risk assessment and prioritization);

Professional identity formation (four cases addressing ethical sensitivity and values);

Technical skill integration (three cases demonstrating evidence-based practice);

Interprofessional collaboration (four cases utilizing SBAR communication frameworks).

Case narratives were reconstructed using (17) three-dimensional narrative analysis method, ensuring alignment with Benner’s clinical competency milestones.

##### 2.2.2.3 Implementation protocol

The intervention comprised biweekly 90-min sessions using a structured four-phase approach: Immersive scenario simulation (25 min): standardized patients reenacted real clinical dilemmas; Narrative deconstruction (30 min): small-group case analysis using guided reflection techniques; Skill application (20 min): care plan development based on simulated scenarios; Reflective integration (15 min): structured reflective writing using Boud’s reflection model (18).

Learning outcomes were systematically tracked through: electronic portfolio documentation (hospital education management system); monthly OSCE assessments evaluating technical skills (40%), clinical judgment (30%), and communication (30%).

### 2.3 Data collection

#### 2.3.1 Nursing student professional identity scale

To evaluate the professional identity of nursing students, the Nurse Professional Identity Scale (Chinese version) (19) was administered to both groups at the end of their clinical practicum. This scale comprises five dimensions: self-efficacy and sense of control, coherence, self-determination, perception of patient and organizational influence, and sense of meaningfulness, totaling 21 items. Each item was scored using a 7-point Likert scale, ranging from “strongly disagree” (1 point) to “strongly agree” (7 points). Dimension scores were calculated as the mean of the corresponding item scores, with higher scores indicating a stronger level of professional identity. The Chinese version of the scale demonstrated a Cronbach’s α coefficient of 0.902 and a content validity of 0.905. In this study, the Cronbach’s α coefficient was 0.817, indicating good reliability. The scale effectively captured changes in students’ professional identity and psychological states before and after the intervention. A total of 82 evaluation forms were distributed, all of which were returned, yielding a response rate of 100%.

#### 2.3.2 Core competency scale for nursing interns

This study also used the Core Competency Scale for Nursing Interns to assess the core competencies of the nursing students during their internship (20). The scale consists of five dimensions with a total of 36 items, including: Good Personal Traits (GPT) (7 items), CNS (13 items), Critical Clinical Thinking (CCT) (5 items), Support and Interpersonal Communication Skills (SICS) (5 items), and Professional Development and Self-improvement Capabilities (PDSC) (6 items). Each item on the scale was rated using a Likert 5-point scale (1 = Strongly Disagree, 5 = Strongly Agree), with higher scores indicating stronger core competencies. The Cronbach’s α coefficient for the scale was 0.856, and the content validity index (S-CVI) was 0.91. This scale effectively assesses the changes in nursing students’ overall competencies during clinical practice, particularly in clinical judgment, communication skills, and professional quality. At the end of the internship, both groups of nursing students were surveyed. A total of 82 evaluation forms were distributed, and all 82 forms were returned, resulting in a 100% response rate.

#### 2.3.3 Internship teaching model evaluation scale

A custom-made nursing intern teaching evaluation form was used, and at the end of the course, both groups of students were asked to evaluate the effectiveness of the clinical teaching model. The evaluation covered seven aspects: improvement in learning interest, development of communication skills, cultivation of clinical thinking, strengthening of knowledge application, enhancement of humanistic care, support for teamwork, and improvement in learning methods. The options were “Yes” and “No.” A total of 82 evaluation forms were distributed and all 82 were returned, resulting in a 100% response rate.

### 2.4 Data analysis

In this study, data entry and statistical analysis were performed using Excel 2007 and SPSS 21.0. First, the Shapiro–Wilk test was conducted on the continuous data to check for normality. For data that followed a normal distribution, independent samples *t*-test was used for group comparisons. If the data did not meet the assumption of normality, the Wilcoxon signed-rank test or Mann–Whitney *U* test was applied. Categorical data were presented as frequencies (percentages), and group comparisons were made using the χ^2^ test or Fisher’s exact test. Furthermore, all test results were adjusted using Bonferroni correction to reduce the risk of false positives from multiple comparisons. Continuous data are presented as mean ± SD. All data processing was performed using SPSS 21.0 (IBM, USA), with *P* < 0.05 considered statistically significant.

## 3 Results

**FIGURE 1:**
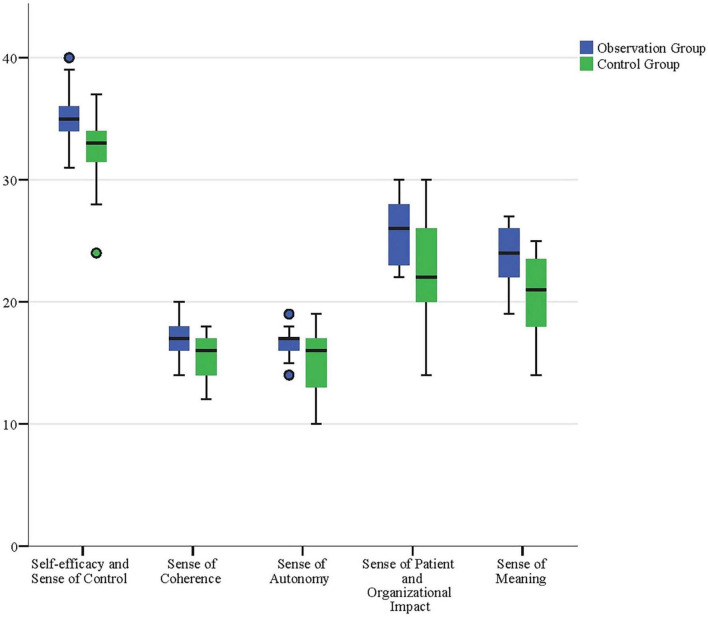
Boxplot comparison of Professional Identity Scale scores across dimensions for observation and control groups post-internship. The boxplot illustrates the distribution of scores across the five dimensions of the Professional Identity Scale, highlighting significantly higher scores in the observation group compared to the control group. These results underscore the positive impact of narrative-based teaching on nursing students’ professional identity.

The primary objective of this study was to evaluate the impact of narrative teaching on the professional identity of vocational nursing interns. By comparing the observation group (*n* = 42) and the control group (*n* = 40), the results demonstrated that the observation group exhibited significant improvements across all dimensions of professional identity. Specifically, the observation group scored significantly higher than the control group in five dimensions: self-efficacy and sense of control, coherence, self-determination, perception of patient and organizational influence, and sense of meaningfulness (*P* < 0.001). These findings suggest that narrative teaching enhances nursing students’ confidence in their clinical abilities (self-efficacy and sense of control), strengthens their sense of professional responsibility and emotional commitment (coherence and perception of patient influence), and effectively promotes their autonomy in clinical practice (self-determination) as well as their passion for the nursing profession (sense of meaningfulness). Among all dimensions, the improvements in self-efficacy and sense of control, as well as sense of meaningfulness, were particularly notable. The mean differences for these two dimensions in the observation group were 2.754 and 3.092, respectively, with 95% confidence intervals excluding zero, indicating highly significant effects of narrative teaching. Additionally, the control group also showed significant improvements in coherence and perception of patient and organizational influence, further validating the effectiveness of narrative teaching in enhancing overall professional identity among nursing interns (Table 1 and Figure 1).

**TABLE 1:** Comparison of scores in each dimension of the Professional Identity Scale after internship between the observation and control groups (scores, *x* ± *s*).

Item	Observation group (*n* = 42)	Control group(*n* = 40)	Mean difference(95% CI)	*P*
Self-efficacy and sense of control	35.43 ± 2.05	32.68 ± 2.46	2.754 (1.759–3.748)	<0.001
Coherence	17.14 ± 1.49	15.45 ± 1.91	1.693 (0.942–2.443)	<0.001
Self-determination	16.55 ± 1.19	15.05 ± 2.44	1.498 (0.642–2.354)	<0.001
Patient and organizational influence	25.86 ± 2.59	22.98 ± 3.83	2.882 (1.435–4.329)	<0.001
Meaningfulness	23.67 ± 2.18	20.58 ± 3.15	3.092 (1.891–4.292)	<0.001

Data are given as mean ± SD or number. CI, confidence interval.

**FIGURE 2:**
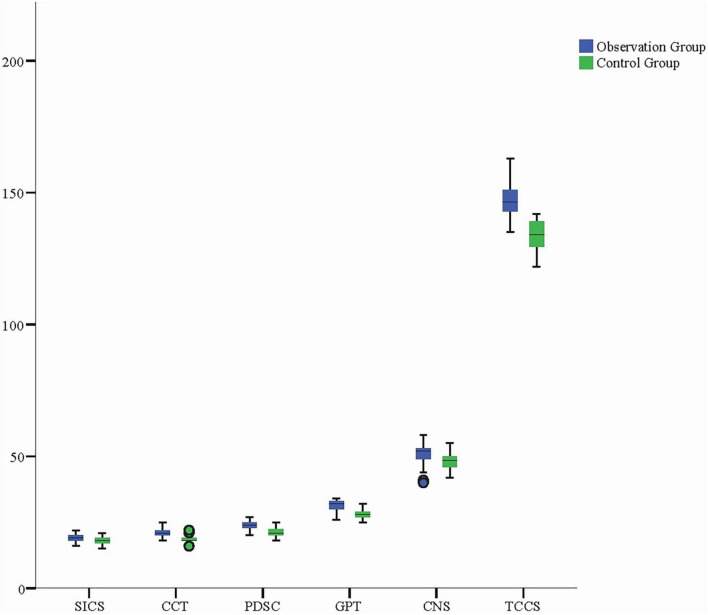
Boxplot comparison of core competency scores across dimensions and total scores for observation and control groups post-internship. The boxplot illustrates the distribution of scores across the dimensions of core competencies, with the observation group achieving significantly higher scores in all areas compared to the control group. These findings highlight the comprehensive benefits of narrative-based teaching in enhancing nursing students’ core competencies. SICS, Support and Interpersonal Communication Skills; CCT, Critical Clinical Thinking; PDSC, Professional Development and Self-improvement Capabilities; GPT, Good Personal Traits; CNS, Clinical Nursing Skills; TCCS, Total Core Competencies Score.

Additionally, the observation group scored significantly higher than the control group in various dimensions, including personal traits, CNS, CCT, support and interpersonal communication abilities, professional development, and overall core competencies (*P* < 0.001). These results indicate that narrative teaching effectively enhanced nursing students’ CNS, personal qualities, and communication and collaboration abilities with others. Among all dimensions, the improvement in personal traits and CNS was particularly notable. The mean differences for the observation group in these two dimensions were 3.496 and 2.880, respectively, with 95% confidence intervals not including zero, indicating that the impact of narrative teaching on these dimensions was highly significant. Furthermore, the control group also showed significant improvements in SICS, as well as PDSC, further validating the effectiveness of narrative teaching in enhancing nursing students’ overall competencies. Notably, the observation group also reached statistical significance in the mean differences for CCT and overall core competencies, with values of 1.221 and 13.111, respectively (95% CI: 1.276–4.484, 10.568–15.653). This indicates that narrative teaching not only promoted nursing students’ clinical thinking abilities but also significantly enhanced their overall core competencies (Table 2 and Figure 2).

**TABLE 2:** Comparison of scores in each dimension and total score of core competencies after internship between the observation and control groups (scores, *x* ± *s*).

Item	Observation group (*n* = 42)	Control group (*n* = 40)	Mean difference (95% CI)	*P*
GPT	31.57 ± 1.71	28.08 ± 1.37	3.496 (2.813–4.179)	<0.001
CNS	50.90 ± 4.21	48.03 ± 2.94	2.880(1.276–4.484)	<0.001
CCT	19.07 ± 1.70	17.85 ± 1.41	1.221 (0.533–1.909)	<0.001
SICS	21.17 ± 1.40	18.50 ± 1.49	2.667 (2.034–3.300)	<0.001
PDSC	24.07 ± 1.76	21.23 ± 1.79	2.846 (2.066–3.626)	<0.001
TCCS	146.79 ± 6.10	133.68 ± 5.43	13.111 (10.568–15.653)	<0.001

Data are given as mean ± SD or number. CI, confidence interval; GPT, Good Personal Traits; CNS, Clinical Nursing Skills; CCT, Critical Clinical Thinking; SICS, Support and Interpersonal Communication Skills; PDSC, Professional Development and Self-improvement Capabilities; TCCS, Total Core Competencies Score.

The observation group demonstrated significantly better performance than the control group across various dimensions, including increased interest in learning, improved communication and expression, developed clinical thinking, strengthened application of knowledge, enhanced humanistic care, supported teamwork, and improved learning methods (*P* < 0.05). These results suggest that narrative teaching not only effectively stimulated nursing students’ learning interest, enhanced communication skills, and cultivated clinical thinking, but also strengthened their awareness of teamwork and humanitarian care. Notably, the observation group scored significantly higher than the control group in learning interest, teamwork, humanitarian care, and improvement in learning methods, with differences showing high statistical significance (*P* < 0.001). These results indicate that narrative teaching not only had a significant impact on enhancing nursing students’ clinical thinking and application of knowledge, but also strengthened their sense of humanitarian care and teamwork abilities. This further demonstrates the comprehensive role of narrative teaching in promoting the overall competence of nursing students (Table 3).

**TABLE 3:** Evaluation of the internship model by the observation and control groups [*n* (%)].

Item	Observation group (*n* = 42)	Control group (*n* = 40)	*P*
Increased learning interest	38 (90.48)	25 (62.50)	0.003
Improved communication and expression	36 (85.71)	22 (55.00)	0.002
Cultivated clinical thinking	37 (88.10)	26 (65.00)	0.013
Strengthened application of knowledge	39 (92.86)	25 (62.50)	0.001
Enhanced humanitarian care	38 (90.48)	21 (52.50)	<0.001
Facilitated teamwork	39 (92.86)	20 (50.00)	<0.001
Improved learning methods	38 (90.48)	23 (57.50)	0.001

## 4 Discussion

The primary objective of this study was to evaluate the effectiveness of narrative teaching in clinical internship education for vocational nursing students. A comparison between the observation and control groups in terms of professional identity and core competencies revealed that narrative teaching significantly enhanced nursing students’ core competencies, particularly in clinical thinking, nursing skills, interpersonal communication, self-development, and professional identity. These findings provide strong support for the application of narrative teaching as an effective educational tool in nursing internship education.

### 4.1 Theoretical significance of the main findings

The findings of this study emphasize the significant impact of narrative teaching as an innovative educational method in enhancing students’ professional identity and clinical core competencies. Narrative teaching not only facilitated students’ knowledge acquisition but, more importantly, helped them strengthen their emotional connection to the nursing profession and sense of responsibility through the storytelling and listening of nursing experiences (21–23). According to Mezirow’s Transformative Learning Theory (24, 25), narrative teaching can prompt students to reflect on their experiences, facilitating a transformation in cognition and attitudes through an emotional learning approach, which in turn influences their professional behavior and career choices.

In this study, the observation group showed significant improvements across various dimensions (such as clinical thinking, communication skills, teamwork, etc.), further validating the positive role of narrative teaching in developing nursing students’ professional identity and CNS. This is consistent with Schön’s theory of reflective practice, which emphasizes the importance of reflection in the development of professional competence and practice (26, 27). Narrative teaching, by promoting reflection and self-awareness among students, enables them to make better decisions and communicate more effectively in clinical settings, thereby enhancing their clinical practice abilities.

Although this study was conducted at a single institution, it is noteworthy that nursing vocational colleges across China uniformly adhere to the National Teaching Standards for Vocational Education (Nursing Programs), which standardizes curriculum design and clinical practice requirements nationwide. Our data reveal significant opportunities for improvement in students’ clinical nursing competencies, aligning with the World Health Organization’s Global Strategic Directions for Nursing and Midwifery 2021–2025, which highlights the “widespread need to enhance clinical decision-making skills among nursing students in Asia.” Despite the inherent limitations of a single-center study, the adoption of stratified random sampling and standardized intervention protocols provides empirical evidence to inform reforms in nursing education within vocational training systems.

### 4.2 Comparison of research findings with existing literature

The results of this study are consistent with existing literature, demonstrating that narrative teaching significantly enhances nursing students’ clinical thinking, communication skills, and teamwork abilities. Zmijewski’s research found that narrative teaching, through an emotional learning approach, helps nursing students integrate theory with practice, thereby strengthening their clinical decision-making and communication skills (28). Our study further validates this finding, particularly in the improvement of clinical thinking and communication skills, where the observation group scored significantly higher than the control group.

In addition, the effectiveness of narrative teaching in enhancing students’ humanitarian care and learning interest aligns with the findings of Hardie’s research (29). Our study demonstrates that narrative teaching, through emotional learning experiences, enhances nursing students’ awareness of patient care and stimulates their learning interest, which is consistent with existing literature.

### 4.3 Practical significance of narrative teaching

From a practical perspective, this study offers a new teaching model for nursing education, particularly in the clinical internship training of vocational nursing students, where narrative teaching has demonstrated significant applied value. Traditional clinical nursing education models tend to focus more on the transmission of skills and theory, with relatively less emphasis on developing students’ professional identity and emotional engagement (30, 31). Narrative teaching, through the telling of real nursing stories, helps students understand the complexity and significance of nursing work, fostering their passion for the profession and sense of responsibility. Through this emotional teaching approach, nursing students not only gain a better understanding of the challenges in nursing practice but also enhance their care for patients, collaboration with teams, and sense of responsibility toward their work.

Additionally, the intervention effect of narrative teaching on core nursing competencies is significant, providing important practical guidance for the future of nursing education. Nursing education during the internship phase should not be limited to technical training but should also emphasize the development of students’ professional emotions and professional qualities. Narrative teaching offers an effective approach to help students form a deeper emotional connection with the nursing profession, thereby enhancing their overall professional competence.

In traditional Chinese culture, great emphasis has always been placed on the concept of “teaching by example” or “leading through personal conduct” (32, 33). Narrative teaching fully embodies this concept, with “leading by example” serving as the vehicle for the story, representing behavioral education, and “teaching through words” occurring through educational dialogue, which represents verbal education. In an era of rapid development in information and internet technologies, direct interpersonal communication has been increasingly neglected. However, face-to-face narrative education, grounded in both teaching by example and verbal instruction, becomes even more important in promoting students’ physical and mental health as well as their moral and faith development (34, 35). Students inherit not only work experience from their teachers but also the spirit of nursing and the qualities that define the nursing profession.

## 5 Limitations of the study

Although this study provides strong evidence for evaluating the effectiveness of narrative classrooms, there are some limitations. First, the sample size in this study was relatively small, focusing solely on nursing students from vocational colleges, and the data were collected from a single hospital, which may limit the generalizability of the findings. To improve the external validity of the research, future studies should expand the sample size, including more institutions and different types of hospitals, to further validate the effectiveness of narrative classrooms in a broader context. Secondly, the intervention period in this study was relatively short, with evaluations conducted only during the clinical internship, preventing an assessment of the long-term effects of the narrative classroom. Future research could adopt a longitudinal design to track students’ career development, clinical performance, and post-graduation employment, evaluating the sustained impact of narrative classrooms on nursing students’ professional careers.

Lastly, this study relied on self-reported questionnaires to assess nursing students’ professional identity and core competencies, which may introduce some subjective bias. Future research could incorporate more objective evaluation methods, such as clinical teacher assessments, patient feedback, and direct observations of clinical performance, to enhance the objectivity and accuracy of the evaluations.

## 6 Future research directions

Future research can be expanded in the following directions. First, the integration of narrative classrooms with other teaching models (such as flipped classrooms, situated learning, etc.) warrants further exploration. The synergistic effects of these teaching methods can be investigated, evaluating their advantages in enhancing nursing students’ clinical skills and overall competencies. Secondly, the interdisciplinary application of narrative classrooms deserves further investigation. Future research could assess the effectiveness of narrative classrooms in other healthcare fields (such as medicine, public health, etc.) to validate its applicability across different disciplines and domains. Finally, long-term follow-up studies represent an important direction for future research, particularly in evaluating the long-term effects of narrative teaching. Future studies should explore the ongoing impact of narrative classrooms at various stages of nursing students’ career development, especially in terms of their professional growth and clinical performance after graduation.

## 7 Conclusion

This study validated the significant effectiveness of narrative classrooms in the clinical internship education of nursing students in vocational colleges. As an innovative teaching model, the narrative classroom, through the sharing of real stories and situational experiences, significantly enhanced the students’ professional identity and core competencies, particularly in clinical thinking, communication skills, and learning interest. This teaching approach breaks through the limitations of traditional nursing education and provides a new path for modern nursing education that balances emotional development with skill enhancement.

## Data Availability

The original contributions presented in this study are included in this article/supplementary material, further inquiries can be directed to the corresponding author.

## References

[1] Yuan J Zeng X Cheng Y Lan H Cao K Xiao S. Narrative medicine in clinical internship teaching practice. *Med Educ Online.* (2023) 28:2258000. 10.1080/10872981.2023.2258000 37722672 PMC10512813

[2] Chen J Yang Y Shen L Zhang X Hu R. Nursing students’ expectations and career preferences before clinical placement in mainland China: A qualitative exploration. *Nurse Educ Pract.* (2023) 67:103552. 10.1016/j.nepr.2023.103552 36669296

[3] Zhao Z Guo J Zhou J Qiao J Yue S Ouyang Y Perceived social support and professional identity in nursing students during the COVID-19 pandemic era: The mediating effects of self-efficacy and the moderating role of anxiety. *BMC Med Educ.* (2023) 23:117. 10.1186/s12909-022-03968-6 36803504 PMC9936494

[4] Liu F Weng H Xu R Li X Zhang Z Zhao K Nursing interns’ attitudes toward, preferences for, and use of diabetes virtual simulation teaching applications in China: National web-based survey. *JMIR Mhealth Uhealth.* (2021) 9:e29498. 10.2196/29498 34499047 PMC8461537

[5] Zhu Z Xing W Liang Y Hong L Hu Y. Nursing students’ experiences with service learning: A qualitative systematic review and meta-synthesis. *Nurse Educ Today.* (2022) 108:105206. 10.1016/j.nedt.2021.105206 34773814

[6] Zhu X Rask M Xu H. First year nursing students’ reflections about developing their verbal nursing skills during their nursing education in China: A qualitative study. *Front Public Health.* (2023) 11:1149512. 10.3389/fpubh.2023.1149512 37213655 PMC10196177

[7] Conle C. An anatomy of narrative curricula. *Educ Res.* (2003) 32:3–15. 10.3102/0013189X032003003 38293548

[8] Brown S Kirkpatrick M Mangum D Avery J. A review of narrative pedagogy strategies to transform traditional nursing education. *J Nurs Educ.* (2008) 47:283–6. 10.3928/01484834-20080601-01 18557318

[9] Bélanger L Porlier M. [Narrative pedagogy in nursing sciences: Learning activities and challenges]. *Rech Soins Infirm.* (2017) 129:52–9. 10.3917/rsi.129.0052 28956411

[10] Ironside P. Narrative Pedagogy: Transforming nursing education through 15 years of research in nursing education. *Nurs Educ Perspect.* (2015) 36:83–8. 10.5480/13-1102 29194131

[11] Lambert K. Teaching belonging in nursing using narrative pedagogy. *J Nurs Educ.* (2024) [Online ahead of print.]. 10.3928/01484834-20240626-02. 39466409

[12] Fuglsang S Bloch C Selberg H. Simulation training and professional self-confidence: A large-scale study of third year nursing students. *Nurse Educ Today.* (2022) 108:105175. 10.1016/j.nedt.2021.105175 34741915

[13] Arveklev S Tengelin E. Learning to teach at a norm-critical clinical learning centre: A Phenomenographic study. *Nurse Educ Today.* (2024) 139:106250. 10.1016/j.nedt.2024.106250 38759338

[14] Xue M Sun H Xue J Zhou J Qu J Ji S Narrative medicine as a teaching strategy for nursing students to developing professionalism, empathy and humanistic caring ability: A randomized controlled trial. *BMC Med Educ.* (2023) 23:38. 10.1186/s12909-023-04026-5 36653810 PMC9850682

[15] Alves S Magalhães C Fernandes A Palmero M Fernandes H. Gerontology and geriatrics in undergraduate nursing education in Portugal and Spain: An integrative and comparative curriculum review. *Healthcare.* (2024) 12:1786. 10.3390/healthcare12171786 39273810 PMC11395543

[16] Aycock D Hayat M. Strategies for the planning and handling of missing data in nursing research. *J Nurs Educ.* (2020) 59:249–55. 10.3928/01484834-20200422-03 32352538

[17] Charon R. Narrative Medicine: A Model for Empathy, Reflection, Profession, and Trust. *JAMA*. (2001) 286:1897–1902. 10.1001/jama.286.15.1897 11597295

[18] Boud D Keogh R Walker D, (editors). *Reflection: turning experience into learning*. London: Kogan Page (1985).

[19] Zhu Y Zhang Y Wong F Kuo S Cheung K Lam M Newly graduated nurses’ stress, coping, professional identity and work locus of control: Results of a cross-sectional study in Shanghai, Hong Kong and Taipei. *J Nurs Manag.* (2022) 30:3406–18. 10.1111/jonm.13801 36176010

[20] Wu C Yan J Wu J Wu P Cheng F Du L Development, reliability and validity of infectious disease specialist Nurse’s Core competence scale. *BMC Nurs.* (2021) 20:231. 10.1186/s12912-021-00757-2 34789255 PMC8596351

[21] Josephsen J. Journal of Nursing Education. Examining grief through art. *J Nurs Educ.* (2020) 59:540. 10.3928/01484834-20200817-14 32865592

[22] Crookes P Else F Lewis P. Signature pedagogies: An integrative review of an emerging concept in nursing education. *Nurse Educ Today.* (2020) 84:104206. 10.1016/j.nedt.2019.104206 31733586

[23] Gandra E da Silva K. Teaching strategies for health advocacy for undergraduate nursing students: A scoping review. *Nurs Educ Perspect.* (2023) 44:92–7. 10.1097/01.NEP.0000000000001085 36652660

[24] Ryan C Cant R McAllister M Vanderburg R Batty C. Transformative learning theory applications in health professional and nursing education: An umbrella review. *Nurse Educ Today.* (2022) 119:105604. 10.1016/j.nedt.2022.105604 36265209

[25] Rojo J Ramjan L George A Hunt L Heaton L Kaur A Applying mezirow’s transformative learning theory into nursing and health professional education programs: A scoping review. *Teach Learn Nurs.* (2023) 18:63–71. 10.1016/j.teln.2022.09.013

[26] Anderson J. Reflection. *ELT J.* (2020) 74:480–3. 10.1093/elt/ccaa039

[27] Contreras J Edwards-Maddox S Hall A Lee M. Effects of reflective practice on baccalaureate nursing students’ stress, anxiety and competency: An integrative review. *Worldviews Evid Based Nurs.* (2020) 17:239–45. 10.1111/wvn.12438 32329238

[28] Zmijewski P Lynch K Lindeman B Vetter T. Narrative medicine: Perioperative opportunities and applicable health services research methods. *Anesth Analg.* (2022) 134:564–72. 10.1213/ANE.0000000000005867 35180174

[29] Hardie P Darley A Langan L Lafferty A Jarvis S Redmond C. Interpersonal and communication skills development in general nursing preceptorship education and training programmes: A scoping review. *Nurse Educ Pract.* (2022) 65:103482. 10.1016/j.nepr.2022.103482 36327590

[30] Zhao Z. Theoretical and practical exploration of work-based learning curriculum with a case study. *Vocat Tech Educ.* (2024) 1:518. 10.54844/vte.2024.0518

[31] Zhao F Kennedy G Cleary S Conduit R Zhang W Fu Q Knowledge about, attitude toward, and practice of complementary and alternative medicine among nursing students: A systematic review of cross-sectional studies. *Front Public Health.* (2022) 10:946874. 10.3389/fpubh.2022.946874 35991045 PMC9386551

[32] Shih Y. Improving the learning in life education for young children aged 3 to 6 years: A review on the research themes of early childhood life education in Taiwan. *Children.* (2022) 9:1538. 10.3390/children9101538 36291473 PMC9600767

[33] Chen L Zhang J Zhu Y Shan J Zeng L. Exploration and practice of humanistic education for medical students based on volunteerism. *Med Educ Online.* (2023) 28:2182691. 10.1080/10872981.2023.2182691 36840966 PMC9970200

[34] O’Farrell C. Religious education in the early years: An irish perspective. *Religions.* (2023) 14:459. 10.3390/rel14040459

[35] Seungbeom S. Analysis of research trends in Christian education: Papers published during COVID-19 (2020.3 - 2022.6). *J Christian Educ Korea.* (2022) 72:97–115. 10.17968/jcek.2022.72.005

